# The Year of the Honey Bee (*Apis mellifera* L.) with Respect to Its Physiology and Immunity: A Search for Biochemical Markers of Longevity

**DOI:** 10.3390/insects10080244

**Published:** 2019-08-07

**Authors:** Martin Kunc, Pavel Dobeš, Jana Hurychová, Libor Vojtek, Silvana Beani Poiani, Jiří Danihlík, Jaroslav Havlík, Dalibor Titěra, Pavel Hyršl

**Affiliations:** 1 Institute of Experimental Biology, Faculty of Science, Masaryk University, Kotlarska 2, 611 37 Brno, Czech Republic; 2 Department of Biology, Institute of Biosciences, Center of Study of Social Insects (CEIS), Sao Paulo State University—UNESP, Avenida 24A, 1515 Bela Vista, Rio Claro 13506-900, Brazil; 3 Department of Biochemistry, Faculty of Science, Palacky University Olomouc, Slechtitelu 27, 783 71 Olomouc, Czech Republic; 4 Department of Food Quality and Safety, Faculty of Agrobiology, Food and Natural Resources, Czech University of Life Sciences, Kamycka 129, 252 63 Prague, Czech Republic; 5 Bee Research Institute, Libcice nad Vltavou 252 66, Czech Republic

**Keywords:** honey bee, immunity, physiology, seasonal changes, longevity

## Abstract

It has been known for many years that in temperate climates the European honey bee, *Apis mellifera*, exists in the form of two distinct populations within the year, short-living summer bees and long-living winter bees. However, there is only limited knowledge about the basic biochemical markers of winter and summer populations as yet. Nevertheless, the distinction between these two kinds of bees is becoming increasingly important as it can help beekeepers to estimate proportion of long-living bees in hives and therefore in part predict success of overwintering. To identify markers of winter generations, we employed the continuous long-term monitoring of a single honey bee colony for almost two years, which included measurements of physiological and immunological parameters. The results showed that the total concentration of proteins, the level of vitellogenin, and the antibacterial activity of haemolymph are the best three of all followed parameters that are related to honey bee longevity and can therefore be used as its markers.

## 1. Introduction

Honey bees (*Apis mellifera* L.) are fundamental for agriculture, playing a crucial role in the plant life cycle as one of the most important pollinators. More than 75% of the 115 leading crop species worldwide are dependent on or at least benefit from animal pollination [1,2]. Bees (*Apoidea*) are responsible for the pollination of approximately 70% of all crop species worldwide [3]. Mainly for this reason, there are serious economic implications when honey bees struggle with pathogens (*Paenibacillus larvae*, *Ascosphaera apis*, viruses, etc.), parasites and pests (*Varroa destructor*, *Nosema* sp., *Aethina tumida*, etc.), and other factors affecting the survival of the colony [4].

There are also still unexplained cases of colony collapse disorder (CCD) [5,6]. Honey bee colony losses are considered to be a multifactorial problem, combining the influence of biotic and abiotic stressors, e.g., nutrition [7], pesticides [8], and climate change [9]. Therefore, knowledge of the physiological and immunological status of honey bee colonies is pivotal in helping beekeepers counteract potential problems and protect honey bees.

The majority of CCD occur during winter, which is the most risky period for honey bee colonies [10,11]. Therefore, it is of the utmost importance to study changes in the honey bee organism during the winter period. Two characteristic populations of honey bees emerge during the year in temperate climates. Whereas the so-called summer generation is short-living with a lifespan of between 15 and 48 days, the winter generation can survive up to 8 months [12]. Short-living honey bees must collect sufficient amounts of nectar, honeydew, and pollen during the summer to have enough supplies to survive unfavourable times when a crop is not available, i.e., winter. Long-living honey bees regulate colony temperature during the winter season and are predestined to initiate brood rearing at its end, when this generation is replaced by a new summer generation [13,14]. The winter population is also exposed to higher ecological pressure of pests and diseases [15,16,17,18].

There are not only behavioural [19] differences between these two honey bee populations but also physiological ones. For example, the titre of juvenile hormone (JH) is elevated [20,21], and the hypopharyngeal glands are hypertrophied in summer bees [22,23]. In contrast, the amount of storage and the antioxidant protein vitellogenin (Vg) is increased in the long-living generation [20,24,25]. Fat body, which is used as a store of nutrients and a source of energy, is also enlarged in winter bees [26]. Moreover, the immune response of honey bees varies over the year as it relies on different mechanisms. Short-living generations combine humoral and cellular immune reactions, whereas long-living populations depend mostly on humoral immunity because the functions of haemocytes during the winter season are limited [27,28,29,30]. Last but not least, honey bees are social insects; a social immunity [31,32,33] and social caste [34] play an important role in colony defence as well.

The successful over-wintering of honey bee colonies depends on the size and quality of the winter bee generation [23,35]. Good beekeeping practice, which is focused mainly on Integrated Pest Management with a special focus on mites [36], can help to protect long-living honey bees. However, the complete biochemical characterisation of winter and summer generations is so far missing. Here, we present the comprehensive results of basic biochemical analyses which could help beekeepers to evaluate the physiological status of their colonies within the whole year and thus help to evaluate the presence of long-living bees in colony. In this study, we followed the selected honey bee colony over a period of approximately two years and monitored changes in the physiology and immunity of honey bees in the hive. We have identified several physiological and immune parameters which significantly differ between summer and winter honey bee populations and we propose these parameters as markers of longevity.

## 2. Materials and Methods

### 2.1. Experimental Bees

Honey bees, *Apis mellifera* L., used in the experiment were obtained from a colony kept in Kývalka apiary near Brno, Czech Republic (GPS location: 49.1886747N, 16.4513211E). The hive chosen for experiments was selected by a professional apiarist with more than 30 years of experience in beekeeping. The experimental colony was selected based on its health and development conditions. The colony was nosema disease and varroasis negative without any clinical symptoms of other diseases. The queen was introduced to the colony in 2015 and remained the same during the whole experiment (March 2017–September 2018), until the queen’s supersedure in October 2018. The colony performed stable brood rearing during years 2015 and 2016. The experimental colony received standard care during the whole experiment. It was treated against varroasis by flumethrin and fed by approximately 15 kg of sucrose solution (3:2 sucrose/water) every autumn; no other supplements were added to the hive. All samples for analysis were collected from the experimental colony to observe the long-term developmental characteristics of the hive and to minimize genetic variance. At the beginning of each month, approximately 150 bees were collected randomly from the colony. Specifically, the bees were shaken off the comb on plastic plate from which they were swept to plastic vials covered by perforated caps to enable ventilation. To avoid the sampling of newly hatched adults, honey bees were collected from the comb which was not part of the brood chamber but was located right next to it. The data gathered from the experimental colony (colony 1) were one-time compared in June 2018 to other two selected colonies (colony 2 and 3) located in the same apiary. All colonies received the same treatment, developed independently, and were led by unrelated queens. Temperature and humidity at the apiary were recorded continuously during the experiment. The average values from 3 days prior collection are plotted in results.

### 2.2. Haemolymph Collection

Bees were quickly transported to the laboratory in plastic vials with perforated cap to enable air flow, and haemolymph samples were collected immediately after arrival by cutting off the abdomen and gently pressing the thorax. The haemolymph samples were always taken within two hours after collecting the bees to plastic vials. Two microliters of haemolymph were bled out from each individual honey bee, and if not stated otherwise, the haemolymph from ten bees was pooled to prepare a mixed sample. Fresh haemolymph samples were used to determinate the concentration of haemocytes, the total protein concentration, and phenoloxidase activity. Haemolymph samples used to determinate the other physiological and immunological parameters were diluted with phenylthiourea solution to prevent melanisation and coagulation. Specifically, 20 µL of collected haemolymph was mixed with 5 µL of phenylthiourea (PTU, 1 mg/mL) in phosphate buffer (PBS, pH 7.0) and stored at −80 °C for further use.

### 2.3. Physiological Parameters

The total protein concentration in haemolymph was measured. Five bees were bled out per sample without the use of PTU solution and five samples were prepared each month of the experiment. The samples were collected on ice to prevent melanisation. The total protein concentration was measured according to the Bradford method using a commercial kit (Bio-Rad, Hercules, CA, USA). The optical density was measured using a Multiscan GO spectrophotometer (Thermofisher Scientific, Waltham, MA, USA) at 700 nm. The exact amount of total protein was calculated from a standard calibration curve prepared from bovine serum albumin (Sigma-Aldrich, St. Louis, MO, USA).

The total concentration of lipids was determined by the sulpho-phosho-vanillin method according to Zollner et al. [37] with modifications by Kodrík et al. [38]. One microliter of 1.25× diluted haemolymph sample was mixed with 200 µL of sulphuric acid (Penta, Prague, Czech Republic) and incubated at 100 °C for 10 min. After quick cooling in a water bath, 2 mL of 13 mM vanillin (Carl Roth Gmbh & Co. KG, Karlsruhe, Germany) in 66.8% phosphoric acid (Penta, Prague, Czech Republic) was added and the resulting solution incubated for 30 min at room temperature. The optical density of samples was measured using a Sunrise spectrophotometer (Tecan, Männedorf, Switzerland) at 546 nm. The concentration of total lipid was calculated from a standard calibration curve prepared from the serial dilution of oleic acid (Penta, Prague, Czech Republic).

The total concentration of carbohydrates was measured using the anthrone method [39]. 50 µL of diluted sample was mixed with 150 µL of 10.3 mM anthrone (Sigma-Aldrich, St. Louis, MO, USA) in 98% sulphuric acid (Penta, Prague, Czech Republic), after which the solution was gently mixed and incubated at 4 °C for 10 min. Then, the mixture was incubated at 100 °C for 20 min and then again at room temperature for 20 min. The optical density was measured using a Sense spectrophotometer (Hidex, Turku, Finland) at 620 nm with agitation prior to reading. The total concentration of carbohydrates was calculated according to a calibration curve prepared from differentially diluted glucose (Sigma-Aldrich, St. Louis, MO, USA).

Levels of vitellogenin (~180 kDa) were determined by sodium dodecyl sulphate (SDS) electrophoresis according to Gätschenberger et al. [40] with modifications using a Miniprotein II apparatus (Bio-Rad, Hercules, CA, USA). Five microliters of frozen haemolymph samples were diluted 50× in 0.125 M Tris buffer pH 6.8, and 10 µL of diluted sample were mixed with 10 µL of sample buffer, 7 µL of mercaptoethanol (Sigma-Aldrich, St. Louis, MO, USA), and 73 µL of 0.125 M Tris buffer pH 6.8, incubated for five minutes at 90 °C, and loaded onto 7.5% acrylamide separation gel. After separation by SDS electrophoresis, the gels were transferred to 1.2 mM Coomassie blue (R250, Bio-Rad, Hercules, CA, USA) staining solution; 4:5:1 water/methanol/acetic acid (Penta, Prague, Czech Republic). Subsequently, gels were destained in a solution of 2 mM acetic acid in 33.3% methanol until the protein bands were clearly visible. Gels were scanned, and the densities of vitellogenin bands were measured in ImageJ v.1.52a (National Institutes of Health, Bethesda, MD, USA). The total vitellogenin concentration was calculated according to a calibration curve prepared from the serial dilution of bovine serum albumin (66.5 kDa, Sigma-Aldrich, St. Louis, MO, USA) that was present on each acrylamide gel.

### 2.4. Cellular Immunity

To determine the concentration of circulating haemocytes, 10 µL of haemolymph from five bees were mixed with 2.5 µL of PTU in PBS (1 mg/mL). The haemocytes were counted using a haemocytometer under a CX 31 light microscope (Olympus, Tokyo, Japan) with phase contrast. Four samples were prepared each month of the experiment. The number of haemocytes was expressed as the count of haemocytes per microliter of haemolymph and evaluated within the experiment.

### 2.5. Humoral Immunity

The antibacterial activity of honey bee haemolymph was determined by radial diffusion assay on agar plates inoculated with *Micrococcus luteus* (CCM 169). Bacteria were cultured overnight in liquid LB medium (MOBIO, Carslbad, CA, USA) on a rotary shaker (100 RPM, room temperature). After cultivation, the suspension was diluted to OD_600_ 1.5, mixed with melted LB agar (4% agar in LB, MOBIO, Carlsbad, CA, USA) in the ratio 1:500 (v/v) at a temperature below 50 °C and then poured on Petri dishes. The agar plates supplemented with *M. luteus* were left to solidify before wells of diameter 2 mm were punctured. The honey bee haemolymph and a serial dilution of pure lysozyme (EC 3.2.1.17; Sigma-Alrich, St. Louis, MO, USA) were loaded into the wells to a volume of 5 μL. After incubation at 30 °C for 24 h, the inhibition zones around wells were scored and the antibacterial activity of haemolymph was expressed as the equivalent of lysozyme standard.

The activity of phenoloxidase (PO) was measured in freshly collected haemolymph without phenylthiourea treatment, as published before [41,42]. Haemolymph from five honey bees per sample (total volume 10 µL) was collected in ice-cold PBS (190 µL; pH 7.0) and gently mixed by pipetting. Forty microliters of diluted haemolymph were transferred to a microplate well and incubated for 5 min at room temperature. After incubation, 160 μL of PO substrate 3,4-dihydroxy-DL-phenylalanine (3 mg/mL in PBS; Sigma-Aldrich, St. Louis, MO, USA) was added to start the reaction. The increase in absorbance at 492 nm was measured every two minutes for 30 min using a Sunrise plate reader (Tecan, Männedorf, Switzerland). PO activity was determined as the melanisation rate after 30 min of reaction and expressed as the integral of the reaction curve.

### 2.6. Data Presentation and Statistical Analysis

The results were plotted using Prism software (GraphPad Software, version 7.0, San Diego, CA, USA). Statistical differences were evaluated by one-way ANOVA with post hoc Dunn’s test comparing two short-living populations, May 2017–August 2017, i.e., summer 2017, and May 2018–August 2018, i.e., summer 2018, and one long-living generation, October 2017–March 2018, i.e., winter 2017/2018. To compare differences only between summer and winter generations, transient months (March, April, September), when a mixture of honey bees were present in hives, were omitted from the analysis. Compilation of statistical data and p-values of Dunn’s test calculated by GraphPad are provided in supplementary material (Tables S1 and S2). The multivariate Spearman correlations test was performed to test relationships between the measured variables (physiological and immunological parameters). The Spearman correlation coefficient *r* varies from −1 to +1. An *r* value close to −1 indicates a negative correlation, while an *r* value close to +1 indicates a positive correlation. As the correlation coefficient value goes towards 0, the relationship between the two variables is considered weaker.

Principal component analysis (PCA) was performed to assess correlations between the physiological parameters (total protein, lipid, carbohydrate and vitellogenin concentrations, antibacterial activity, phenoloxidase activity, and haemocyte concentration) and the seasons when the workers were collected (summer 2017, winter 2017/2018, and summer 2018). A statistical test was performed on data using the free PAST 3.05 software (University of Oslo, Oslo, Norway). Since the variables are in different units of measurements, we used a correlation matrix to standardize each of the variables and to perform PCA. In the graph, summer 2017 is represented by full black dots, winter 2017/2018 by blue squares, and summer 2018 by red crosses.

## 3. Results

### 3.1. Physiological Parameters

**Figure 1:**
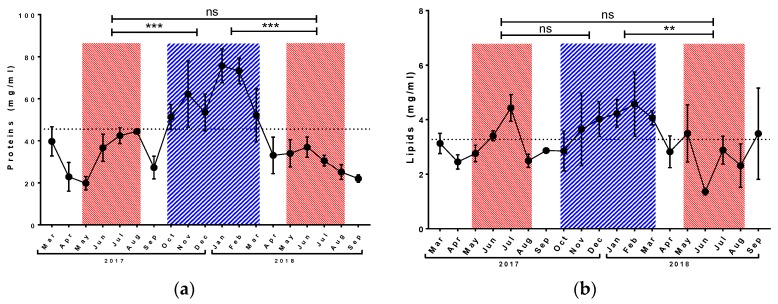

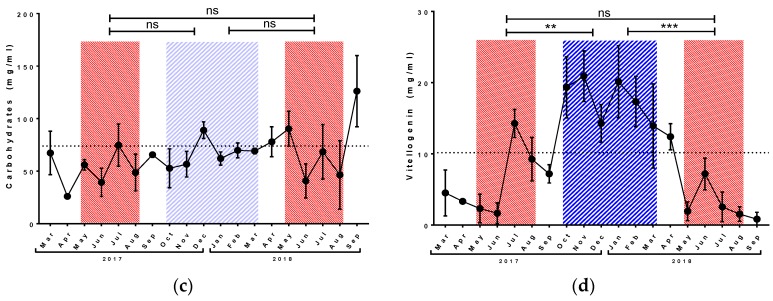
Seasonal development of total protein (**a**), lipid (**b**), and carbohydrate (**c**) concentration and level of vitellogenin (**d**) in honey bee haemolymph. Red striped areas indicate summer generations of honey bees (May–August), and blue area indicates winter generations (October–March). Dots represent mean ± SD, n = 3–5. Dotted line represents annual average (October 2017–September 2018). Asterisk indicates significant difference ** *p* < 0.01, *** *p* < 0.001, ns = not significant.

The concentration of total proteins in haemolymph varied around 30 mg/mL during both tested summer periods, and we did not observe any differences between the two corresponding short-living honey bee populations investigated in the experiment (Figure 1a). However, the protein level increased significantly during the winter period and remained at over 50 mg/mL from October to March.

During both tested summer periods, the concentration of total lipid oscillated around the annual average, with a peak of 4.4 ± 0.5 mg/mL in July 2017 (Figure 1b) and a minimum of 1.3 ± 0.1 mg/mL in June 2018. The short-living honey bees did not differ from each other, but honey bees collected in summer 2018 differed significantly from individuals collected in winter 2017/2018. The level of lipids in winter was over 4 mg/mL.

The total carbohydrate concentration was very stable during all studied seasons (Figure 1c), and measured values oscillated around the annual average (73.8 mg/mL) with the exception of the value measured in September 2018. There were no significant differences among all tested seasons.

The concentration of vitellogenin was higher in winter 2017/2018 in comparison with both summer periods (Figure 1d). In the long-living population, the level of vitellogenin was around 20 mg/mL. In both summer seasons, values remained below the annual average of 10.2 mg/mL with the exception of the value 14.2 ± 1.9 mg/mL measured in July 2017. Short-living generations did not differ among themselves. The vitellogenin level in drones was below the limit of detection for particular method (Figure S1). The result was confirmed by western blot analysis (Figure S2).

### 3.2. Immunological Parameters

**Figure 2:**
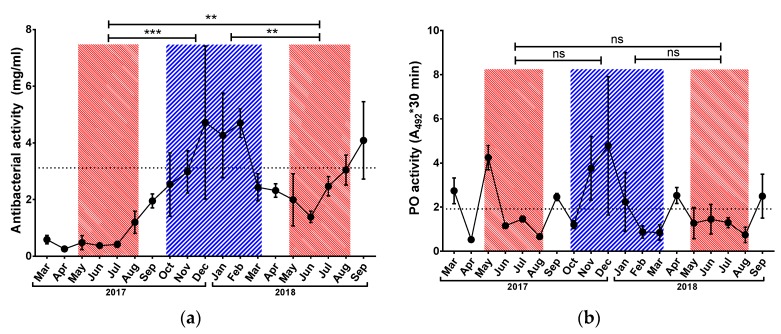

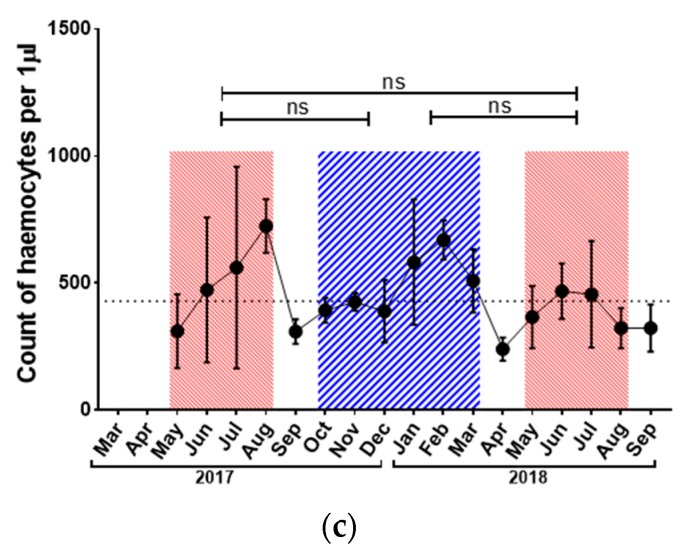
Seasonal development of antibacterial activity (**a**), the activity of phenoloxidase (**b**) and haemocytes counts (**c**) in honey bee haemolymph. Red striped areas indicate summer generations of honey bees (May–August), and blue area indicates winter generation (October–March). Dots represent mean ± SD, n = 3–5. Dotted line represents annual average (October 2017–September 2018). Asterisk indicates significant difference ** *p* < 0.01, *** *p* < 0.001, ns = not significant.

Antibacterial activity (Figure 2a) varied around 4 mg/mL in the winter season, with a peak of 4.7 ± 2.7 mg/mL in December 2017. In both tested short-living generations, the level of antibacterial activity was lower than the annual average. Values in summer 2018 reached around 2 mg/mL and were significantly higher than values in summer 2017, which reached 0.5 mg/mL. Both short-living generations showed significantly lower antibacterial activity than the winter population.

Phenoloxidase activity increased slightly but not significantly in the winter period (Figure 2b). This measured parameter showed a high degree of variability and no statistically significant differences were detected among the monitored seasons.

In contrast with PO activity, the concentration of haemocytes was very stable during the monitored period (Figure 2c). Haemocyte counts oscillated around 500 cells/µL, which was very close to the annual average (429 cells/µL). There were noticeable increases in summer 2017 and winter 2017/2018, with values around 600 cells/µL, while the summer 2018 value was around the annual average. Statistical analysis did not reveal any differences in haemocyte concentration dependent on the season.

The experimental colony (colony 1) was compared to other two colonies (colony 2 and 3) in June 2018 to address the variability of measured parameters (Figures S3 and S4). No significant differences were observed among tested colonies except of PO activity which was lower in colony 1 than in both other tested colonies (Figure S4b).

**Figure 3:**
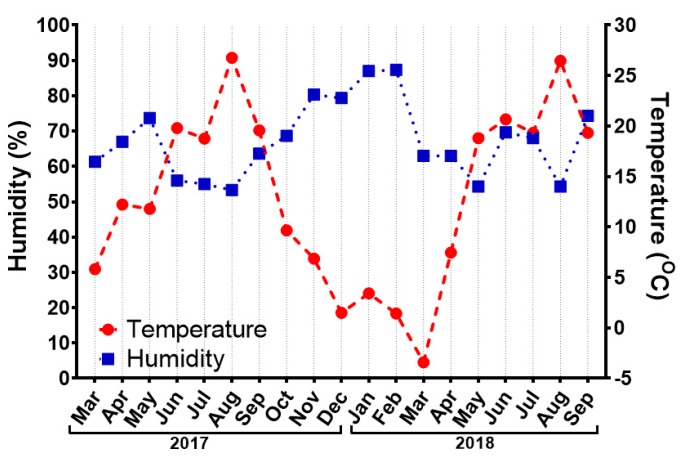
Changes in humidity (blue squares, left *y*-axis) and temperature (red dots, right *y*-axis) at the location of the apiary where honey bees were collected. Each point represents the mean value of measurements three days before the collection and on the day of collection.

In parallel with physiological and immunological parameters of honey bees, we followed changes in temperature and humidity at the site of the apiary where honey bees were collected (Figure 3). The highest recorded temperatures were 26.7 °C and 26.4 °C in August 2017 and August 2018, respectively. The lowest temperature was −3.4 °C measured in March 2018. Relative humidity oscillated between 50% and 90%, with the highest values in January and February 2018.

Correlations among all measured physiological and immunological parameters were identified using the Spearman correlation test; significant results from all three seasons are summarised in Table 1. Total protein concentration correlated positively with antibacterial activity both in summer 2017 and winter 2017/2018, with r_s_ values of 0.648 and 0.542, respectively. During summer 2017, protein concentration correlated positively with vitellogenin concentration (r_s_ = 0.608) but negatively with phenoloxidase activity (r_s_ = −0.81). Moreover, total lipid concentration correlated positively with carbohydrates (r_s_ = 0.771) in summer 2018 and with haemocyte count in winter 2017/2018 (r_s_ = 0.471).

**Figure 4:**
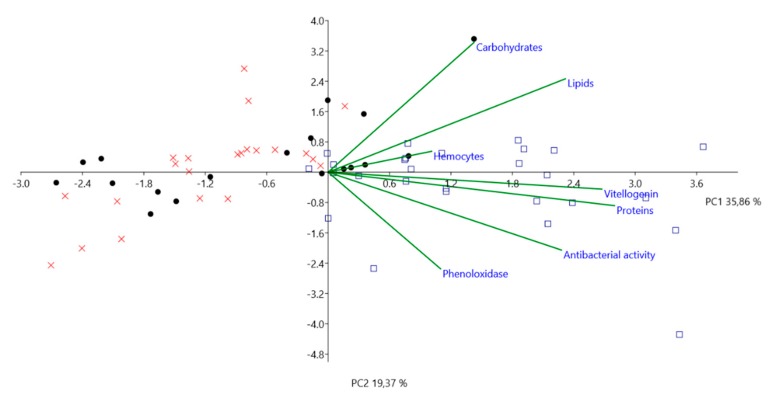
Principal component analysis of variances (proteins, lipids, carbohydrates, vitellogenin, antibacterial activity, phenoloxidase, and haemocytes). Component 1 (PC1) depicted as the *x*-axis explains 35.86% of sample variability. Component 2 (PC2) depicted as the *y*-axis explains 19.37% of sample variability. Black dots, red crosses, and blue squares represent values for summer 2017, summer 2018, and winter 2017/2018, respectively. Green lines express the extent to which each variable contributes to the explanation of the differences.

In the present study, the three principal components generated by PCA (PC1, PC2, and PC3) explained 71.67% of the variability among the groups (summer 2017, winter 2017/2018, and summer 2018). PCA separated the workers from summer 2017, summer 2018, and winter 2017/2018 into two major groups and correlated them with the physiological parameters (Figure 4). Summer 2017 and summer 2018 are clearly grouped together mainly on the left side of the graph, while winter 2017/2018 is on the right side. All variables are on the right side of the graph, which means that they act together to separate summer from winter bees. However, each variable has its own significant value. The separation of summer and winter groups is due to the most significant variables in the data, their significance given by their factor loading, which ranges from +1 to −1. There were two major significant variables, proteins and vitellogenin, with factor loadings of 0.811 and 0.775, respectively. Lipids (factor loading 0.672) and antibacterial activity (factor loading 0.661) were also significant variables, though weaker than proteins and vitellogenin. Haemocytes, phenoloxidase, and carbohydrates exerted less influence on summer and winter groups in comparison with the other parameters.

**Figure 5:**
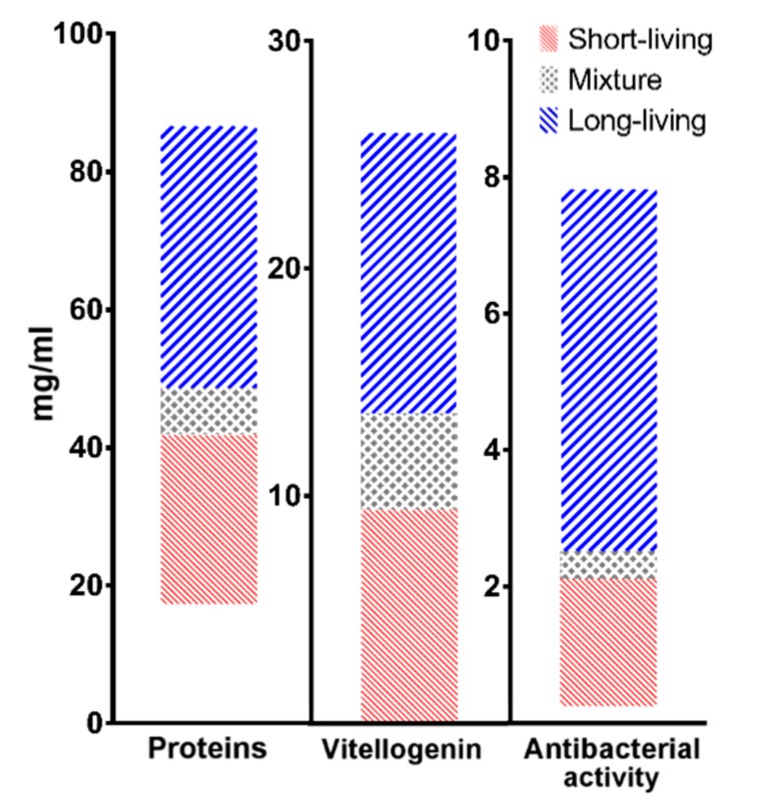
Graphical depiction of proposed physiological ranges in protein, vitellogenin, and antimicrobial level for each season. Red, blue, and grey areas represent short-living, long-living, and mixed populations, respectively.

Range of the values per each population for the three selected markers of longevity based on PCA analysis were depicted as 75% percentile of the values (Figure 5). All of the values were divided into quartiles; upper and lower quartiles in summer and winter populations were cut off, respectively. These values were considered as the mixture of both populations or might be negatively affected by unspecified environmental factors, i.e., pathologies, and therefore were excluded from the range. Total concentration of proteins in short-living population ranged between 17 and 42 mg/mL, and its level was elevated in long-living populations to 49–87 mg/mL. Similarly, level of vitellogenin was lower in summer generation (0–10 mg/mL) in comparison to winter generation (14–26 mg/mL). The antibacterial activity, measured as equivalent of lysozyme concentration, decreased in short-living honey bees 0–2 mg/mL and increased in long-living bees. The physiological ranges of tested parameters for each population are summarized in Table 2.

## 4. Discussion

Although it has been known for a long time that two distinct honey bee populations exist in the same colony, such knowledge based on observations of lifespan [12], knowledge concerning their biochemical parameters in general, is limited. Mostly, vitellogenin, as a storage and antioxidative protein, is associated with honey bee longevity [13,20,26,27,43,44]. However, other important parameters that may also characterise distinct populations of honey bees have received less attention. In an attempt to fill this gap and improve knowledge about longevity, the main purpose of the present study was to evaluate all fundamental physiological and immunological parameters of adult workers and correlate them with long-living and short-living generations of honey bees. In accord with published literature [13], we divided the populations of honey bees according to seasonal development. Bees from October to March were considered to belong to long-living population and bees from the rest of the year as belonging to short-living population. September and April were considered as transition months between seasons and less attention was given to them, since there was a high probability of an even distribution of both honey bee populations in the hive.

According to previous observations, higher contents of proteins and lipids in the haemolymph of winter bees coincided with higher amounts of these compounds in their fat body [45]. This increase is expected because honey bees have to create storage nutrients for the winter season. All organisms including bees have to maintain stable concentrations of basic nutrients in their circulatory systems and, in case of need, these nutrients can be replenished from storage tissues. In accordance, we found levels of carbohydrates to be very stable during the year. Not surprisingly, we detected a rapid increase in carbohydrates in September 2018, when sampling was conducted the day after the administration of sucrose solution to the beehives as a replacement for honey. As this practice might interfere with evaluation of the seasonal development of carbohydrate level as well as the levels of other nutrients in haemolymph, it clearly shows that sampling for the evaluation of the suggested biochemical parameters of longevity must be planned precisely in regard to colony treatment. During the year, the only sources of proteins and carbohydrates for bees are pollen and nectar, respectively [46]. The differences between summer 2017 and summer 2018 could have resulted from the variance of food sources during the season [47].

Because the cellular part of the immune system in long-living generations was observed to be limited, impaired phagocytosis and nodule formation [27,28], honey bees have to rely on the humoral part of the immune system during the winter period. This corresponds with the higher antibacterial activity of haemolymph in the winter population, which can be caused by inducible antibacterial peptides such as apidaecin, hymenoptaecin, abaecin, or constitutive antibacterial compounds such as lysozyme present in haemolymph [10,48,49,50]. Moreover, this increase in antibacterial activity correlates with higher protein levels in the winter honey bee generation. On the other hand, some authors reported reduced expression of immune genes in long-living generation and therefore higher susceptibility to diseases [27,28,51]. Another important part of the humoral immune system—the activity of enzyme phenoloxidase [52]—did not show significantly higher activity during winter. However, we observed a small increasing trend in PO activity in the long-living generation consistent with Lourenço et al. [53], who also showed an increase in PO activity in longer living workers. The honey bee genome possesses only one gene coding the PO precursor prophenoloxidase, whereas three and nine are present in *Drosophila* and *Anopheles*, respectively [49]. Furthermore, PO enzymatic activity is difficult to determine because of the rapid activation and depletion of the PO system [52]. This dynamic process leads to highly variable results, and it is necessary to use freshly collected haemolymph samples. Due to these reasons, the parameter is unsuitable for the occasional testing of honey bee longevity in beekeeping praxis.

During the winter season, honey bees rely on honey storage as the main and only source of food. This diet, composed mainly of carbohydrates, can influence the composition and function of haemocytes in long-living honey bee populations [28,54] leading to the suppression of cellular immunity. During winter, haemocytes participate less in the fight against pathogens than during the summer season. As one of the main reasons, the high energetic costs of haemocytes maintenance was identified in various organisms [55,56,57], which requires many resources that are subdued in winter. Moreover, many authors described that the number of haemocytes in circulation depends on age of the honey bee, and the strong reduction in haemocyte numbers was observed in old bees [24,25,29,30]. Our results showed a slight increase in the number of haemocytes during summer 2017 and winter 2017/2018 in comparison with the annual average but without any statistical significance. Although, we were not able to confirm seasonal-dependent variability of haemocyte numbers, this might partially be caused by sampling the bees of unspecific age.

Vitellogenin is a known internal storage glycolipoprotein synthesized in fat body cells and released into haemolymph [58,59]. Previous studies, in addition to the present one, have considered a higher level of vitellogenin in haemolymph to be a marker of winter bees [43,60,61,62,63]. Because higher amounts of vitellogenin in haemolymph indicate the existence of long-living honey bees, it is considered to be a major component important for overwintering [64]. To confirm that the most abundant 180 kDa form of vitellogenin is present in haemolymph [58,59], we used drone haemolymph sample as negative control. As it was previously described, the drones have the vitellogenin only in the early stages of their life, and it disappears as they are three or more weeks old [40,65,66,67,68]. Our results confirmed these findings; however, the use of only one parameter measurement as a marker of longevity may not be sufficient. According to principal component analysis, the total level of haemolymph proteins correlated highly with physiological changes in the honey bee body during the winter season as well as with antibacterial activity measured against the non-pathogenic model bacteria *M. luteus*. Our conclusions are supported by previous studies [12,63,69,70]. Dainat et al. (2012) reported upregulation of immune genes as predictive marker; concurrently, they mentioned *Varroa* infestation as predictive marker of colony collapse disorder [10].

Because some authors described physiological differences in between honey bees reared in distinct conditions [71] and even variations among colonies [72], we employed measurements of two more colonies in June 2018. All tested hives did not differ in any physiological parameters (Figure S3). Similarly, there is consistency among the colonies in measured immunological reactions (Figure S4), except for PO activity which was lower in colony 1 than in others. However, the PO activity is a highly variable parameter and was not found to be suitable for comparison of summer and winter honey bee populations.

It is known that the weather can have significant influence on honey bee over wintering [73], foraging activity [74] or viral loads [75] in hives. Instead of direct weather impact on honey bee individuals, reduced pollen flow and therefore impaired nutrition can cause problems connected with malnutrition [7,76,77]. Hence, we collected data about weather, specifically temperature and humidity at the apiary location. During the experiment, the changes of weather parameters were typical for temperate climate as characterised especially by low winter temperatures. Minor differences were observed between years 2017 and 2018. However, for instance, the shift of temperature in April 2017 compared to April 2018, where the average temperature was under 10 °C in the latter, is supposed to have an impact on colony development during the rest of the year. Similarly, almost twice as high temperature in May 2018 compared to May 2017 could impact onset of brood rearing [78,79,80] or phenology [81,82,83] of the bees in the colony.

The advantage of selected methods is relative simplicity; therefore, the beekeepers could cooperate with a broad spectrum of laboratories in which the analyses can be implemented. However, if the longer transport and storage time of bees or sampled haemolymph would be considered, the influence of these factors on analyses results should be considered. Regarding the results of our study, the total protein concentration and the levels of vitellogenin and antibacterial activity in haemolymph could be used as relatively simple markers to determine the presence of long-living honey bees in a colony. Based on the results of analyses and their comparison to physiological values provided in Figure 5, beekeepers could adjust the care of their colonies. They can take practical precautions to protect beehives. For example, uniting of two or more weak colonies to create strong one is a possible solution, or several layers of insulation added to a beehive could be used for better protection of underdeveloped colonies [23,35]. Moreover, treatment of short-living populations against pathogens could be reflected in damaged long-living population. Therefore, underdeveloped colonies could be eliminated to save money for feeding and other costs. These precautions based on analyses results might reduce winter loses of honey bees.

The potential of selected markers to distinguish between summer and winter honey bee populations in the temperate climate was demonstrated in current study. Since this study was performed on one hive, future experiments would involve efforts to measure all three suggested parameters on a large number of honey bee colonies to differentiate genotype and environmental variables and correlate the results of analyses to the success overwintering rate. Based on this study, we suggest two preventive samplings per year in June and October. When compared between each other and with data provided in our study, the results of preventive samplings might provide useful insight into the readiness of long-living bees which are indispensable for successful overwintering. The ability to reliably distinguish the two honey bee populations can provide a strong and useful tool for beekeepers. They could estimate proportion of long-living bees in the hives and based on the results predict potential overwintering problems. Our work provides basic knowledge about changes in physiological and immunological parameters in established honey bee colonies over a long time period of almost two years. The data collected can be used as a cornerstone for further studies estimating the proportions of long-living honey bees in their colony.

## 5. Conclusions

This study compared differences between long-living and short-living populations of honey bees. In detail, we described differences in the physiological and immunological parameters of both populations. Winter honey bees had higher amounts of total proteins, lipids, and vitellogenin along with higher levels of antibacterial activity in their haemolymph compared to summer bees. On the basis of this knowledge and in accordance with the results of PCA, we propose levels of total proteins, antibacterial activity, and vitellogenin as markers of long-living honey bee populations. Our results can help beekeepers to confirm the presence of long-living bees in colonies.

## Figures and Tables

**Table 1:** Spearman rank correlations for the different variables which showed significant *p*-values for the three seasons, summer 2017, winter 2017/2018, and summer 2018.

Summer 2017			
Variable	by Variable	Spearman r	*p* Value
Antibacterial activity	Proteins	0.648	0.026
Proteins	Phenoloxidase	−0.81	0.022
Proteins	Vitellogenin	0.608	0.04
**Winter 2017/2018**			
**Variable**	**by Variable**	**Spearman r**	***p*** **Value**
Lipids	Haemocytes	0.471	0.042
Antibacterial activity	Proteins	0.542	0.017
**Summer 2018**			
**Variable**	**by Variable**	**Spearman r**	***p*** **Value**
Lipids	Carbohydrates	0.771	0.001

**Table 2:** Summarization of proposed physiological range for each selected longevity markers based on PCA analysis.

	Short-Living	Long-Living
Proteins	17–42 mg/mL	49–87 mg/mL
Vitellogenin	0–10 mg/mL	14–26 mg/mL
Antibacterial activity	0–2 mg/mL	2.5–8 mg/mL

## Supplementary Materials

The following are available online at https://www.mdpi.com/2075-4450/10/8/244/s1, Figure S1: Gel electrophoretic analysis of vitellogenin concentration; Figure S2: Western blot analysis of vitellogenin; Figure S3: Comparison of physiological parameters; Figure S4: Comparison of immunological parameters; Table S1: Compilation of statistical data; Table S2: *p*-Values of Dunn’s test.
