# Supplementary material for: The Year of the Honey Bee (Apis mellifera L.) with Respect to Its Physiology and Immunity: A Search for Biochemical Markers of Longevity
Source: Insects. 2019 Aug 7;10(8):244. doi: 10.3390/insects10080244 (PMC6723739; doi:10.3390/insects10080244)
Supplement: Supplementary file 1 [file insects-10-00244-s001.pdf]

*insects*

*Article*

# The Year of the Honey Bee (*Apis mellifera* L.) with Respect to Its Physiology and Immunity: A Search for Biochemical Markers of Longevity

**Martin Kunc [1], Pavel Dobeš [1], Jana Hurychová [1], Libor Vojtek [1], Silvana Beani Poiani [1,2], Jiří Danihlík [3], Jaroslav Havlík [4], Dalibor Titěra [5] and Pavel Hyršl [1,*]**

1 Institute of Experimental Biology, Faculty of Science, Masaryk University, Kotlarska 2, 611 37 Brno, Czech Republic
2 Department of Biology, Institute of Biosciences, Center of Study of Social Insects (CEIS), Sao Paulo State University—UNESP, Avenida 24A, 1515 Bela Vista, Rio Claro 13506-900, Brazil
3 Department of Biochemistry, Faculty of Science, Palacky University Olomouc, Slechtitelu 27, 783 71 Olomouc, Czech Republic
4 Department of Food Quality and Safety, Faculty of Agrobiology, Food and Natural Resources, Czech University of Life Sciences, Kamycka 129, 252 63 Prague, Czech Republic
5 Bee Research Institute, Libcice nad Vltavou 252 66, Czech Republic
* Correspondence: hyrsl@sci.muni.cz; Tel.: +420-549-494-510

Received: 27 June 2019; Accepted: 2 August 2019; Published: date

## Supplymentary Materials

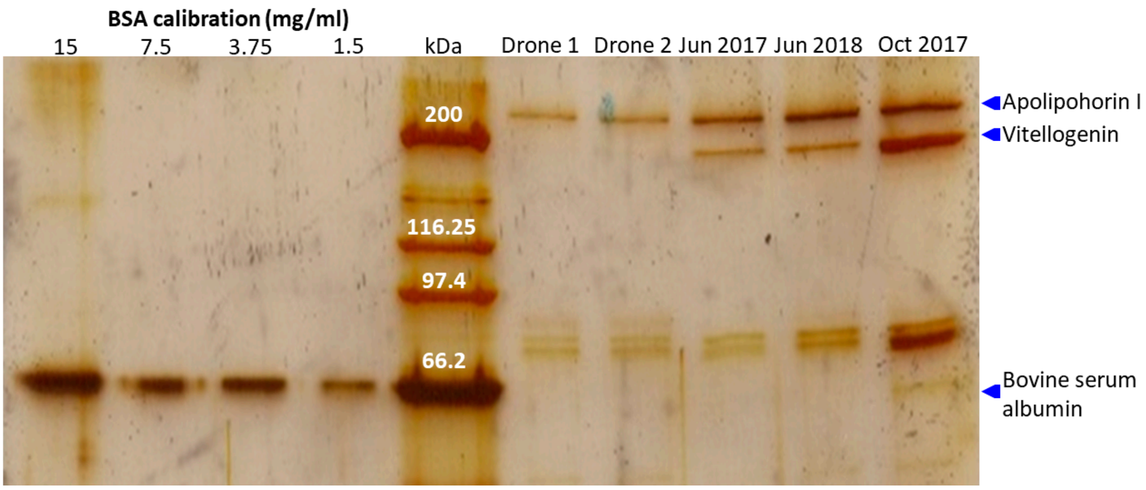


**Figure S1.** To double-check vitellogenin bands identified at Coomasie stained gels, some gels were stained in parallel by silver according to Kirkeby *et al.* [1]. Gel electrophoretic analysis of vitellogenin concentration in drones (Drone 1, 2) and honey bee workers from summer season (Jun 2017, 2018) and winter season (Oct 2017). Bovine serum albumin was used as calibration to calculate concentration of vitellogenin. Size of proteins used in ladder (SDS-PAGE Molecular Weight Standard, Bio-rad, 161-0317, USA) is indicated in the figure.

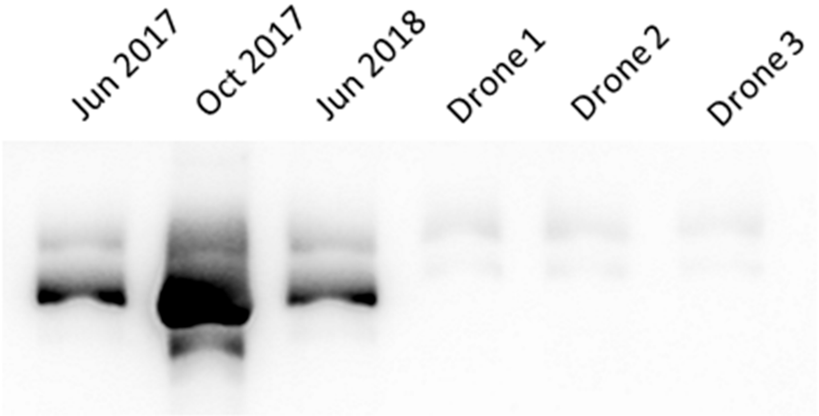


**Figure S2.** Western blot analysis of vitellogenin level in haemolymph of summer/winter population compared to drone haemolymph. For western blot, haemolymph samples were diluted 100×. Diluted samples were subjected to SDS-PAGE and electrotransfered onto Immobilon-P Membrane (Millipore) and immunodetected using primary antibody (Vitellogenin, kindly provided by RNDr. Dalibor Kodrík, CSc., Institute of entomology, Biology Centre CAS, 1:5000) and secondary antibody (Sigma-Aldrich, A0545, 1:5000). The blots were visualized by Immobilon Western Chemiluminescent HRP Substrate (Millipore, USA) in western blot imagining system Fusion SL (Vilber, France).

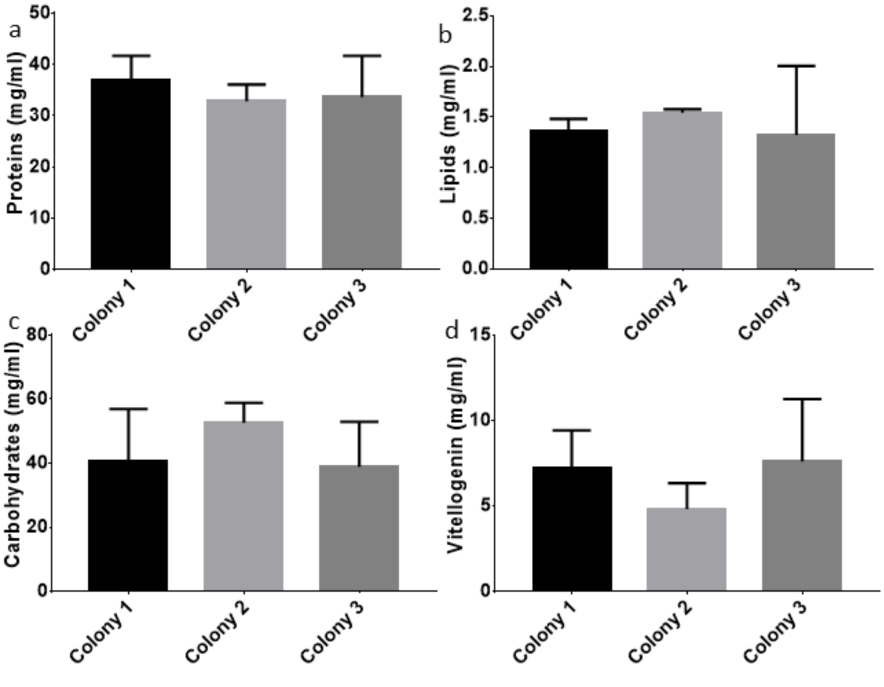


**Figure S3.** Comparison of physiological parameters: total concentration of proteins (**a**), lipids (**b**), carbohydrates (**c**) and vitellogenin (**d**) among experimental bee hive (Colony 1) and two different bee hives (Colony 2 and 3). The measurement was performed in June 2018. No significant difference was observed at the *p*-value 0.05. Columns indicate mean + SD, $n = 3–6$.

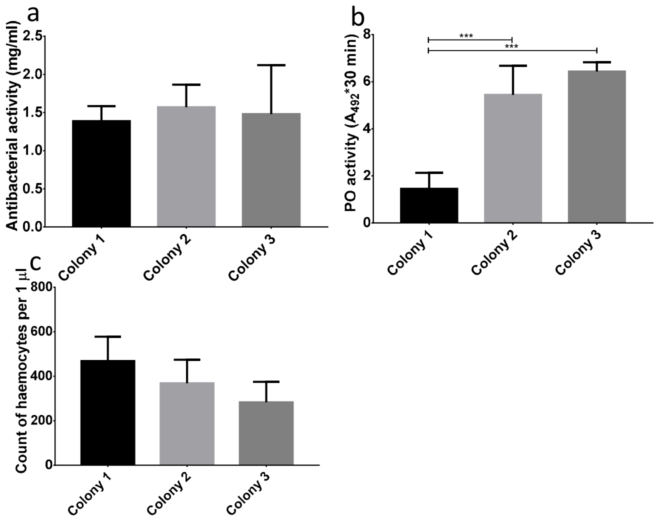


**Figure S4.** Comparison of immunological parameters: antibacterial activity (**a**), phenoloxidase activity (**b**) and count of haemocytes (**c**) among experimental bee hive (Colony 1) and two different bee hives (Colony 2 and 3). The measurement was performed in June 2018. Columns indicate mean + SD, $n$ = 3–6. Asterisk indicates significant difference *** $p < 0.001$, ns = not significant.

**Table S1.** Compilation of statistical data for each tested season. The table contain n—sample size, mean, median, SD—standard deviation and SE—standard error for every tested parameter.

| | **Summer 2017** | | | | | **Winter 2017/2018** | | | | | **Summer 2018** | | | | |
|---|---|---|---|---|---|---|---|---|---|---|---|---|---|---|---|
| | **n** | **Mean** | **Median** | **SD** | **SE** | **n** | **Mean** | **Median** | **SD** | **SE** | **n** | **Mean** | **Median** | **SD** | **SE** |
| **Proteins** | 12 | 35.83 | 40.33 | 10.7 | 3.089 | 27 | 61.95 | 62.4 | 13.77 | 2.65 | 19 | 31.69 | 32.49 | 6.297 | 1.445 |
| **Lipids** | 13 | 3.513 | 3.098 | 1.405 | 0.389 | 19 | 3.916 | 4.044 | 0.897 | 0.206 | 15 | 2.512 | 2.554 | 0.969 | 0.25 |
| **Carbohydrates** | 13 | 56.94 | 50.21 | 19.42 | 5.387 | 22 | 64.96 | 67.64 | 14.48 | 3.087 | 15 | 59.91 | 57.39 | 29.78 | 7.689 |
| **Vitellogenin** | 13 | 8.024 | 8.384 | 5.705 | 1.582 | 19 | 17.78 | 17.21 | 4.648 | 1.066 | 15 | 3.015 | 2.439 | 2.651 | 0.684 |
| **Antibacterial activity** | 13 | 0.665 | 0.459 | 0.435 | 0.121 | 19 | 3.64 | 3.353 | 1.557 | 0.357 | 15 | 2.349 | 2.526 | 0.795 | 0.205 |
| **PO activity** | 8 | 1.88 | 1.3 | 1.507 | 0.533 | 22 | 2.38 | 1.303 | 2.11 | 0.449 | 22 | 1.183 | 1.165 | 0.551 | 0.118 |
| **Haemocytes** | 18 | 494.8 | 412.5 | 288.5 | 67.99 | 24 | 495.6 | 431.3 | 154.1 | 31.46 | 16 | 403.9 | 393.8 | 139 | 34.76 |

**Table S2.** *p*-Values of Dunn´s test calculated by GraphPad software. The table contain *p*-values for comparison of every season.

| | **Summer 2017 × Winter 2017/2018** | **Summer 2017 × Summer 2018** | **Summer 2018 × Winter 2017/2018** |
|---|---|---|---|
| **Proteins** | <0.001 | 0.94 | <0.001 |
| **Lipids** | 0.42 | 0.20 | 0.001 |
| **Carbohydrates** | 0.65 | >0.99 | >0.99 |
| **Vitellogenin** | 0.002 | 0.27 | <0.001 |
| **Antibacterial activity** | <0.001 | 0.003 | 0.12 |
| **PO activity** | >0.99 | >0.99 | 0.30 |
| **Haemocytes** | >0.99 | >0.99 | 0.31 |

## Reference

1. Kirkeby, S.; Moe, D.; Bòg-Hansen, T.C. The silver staining procedure of sodium dodecyl sulfate-gels may be accelerated by shortening fixation time. *Electrophoresis* **1993**, *14*, 51–55.
